# Supplementary material for: The influence of canopy radiation parameter uncertainty on model projections of terrestrial carbon and energy cycling
Source: PLoS One. 2019 Jul 18;14(7):e0216512. doi: 10.1371/journal.pone.0216512 (PMC6638863; doi:10.1371/journal.pone.0216512)

Supporting Information for

The influence of canopy radiation parameter uncertainty on model projections of terrestrial carbon and energy cycling

Authors: T. Viskari^1,4^, A. Shiklomanov^2,3^, M.C. Dietze^3^ and S.P. Serbin^1^

1. Brookhaven National Laboratory, Upton, New York, USA
2. Joint Global Change Research Institute, Pacific Northwest National Laboratory, College Park, MD
3. Department of Earth & Environment, Boston University, Boston, Massachusetts, USA
4. Finnish Meteorological Institute, Helsinki, Finland

**Contents of this file**

Supplemental Figure 1

**Introduction**

Elasticity and output standard deviations standardized by the median with regard to radiative parameter uncertainties in the ED2 model. These results are based on the same analysis as explained in the manuscript. The figures are presented here to save space in the manuscript while the results here are more straightforward to explain just in text.

A)


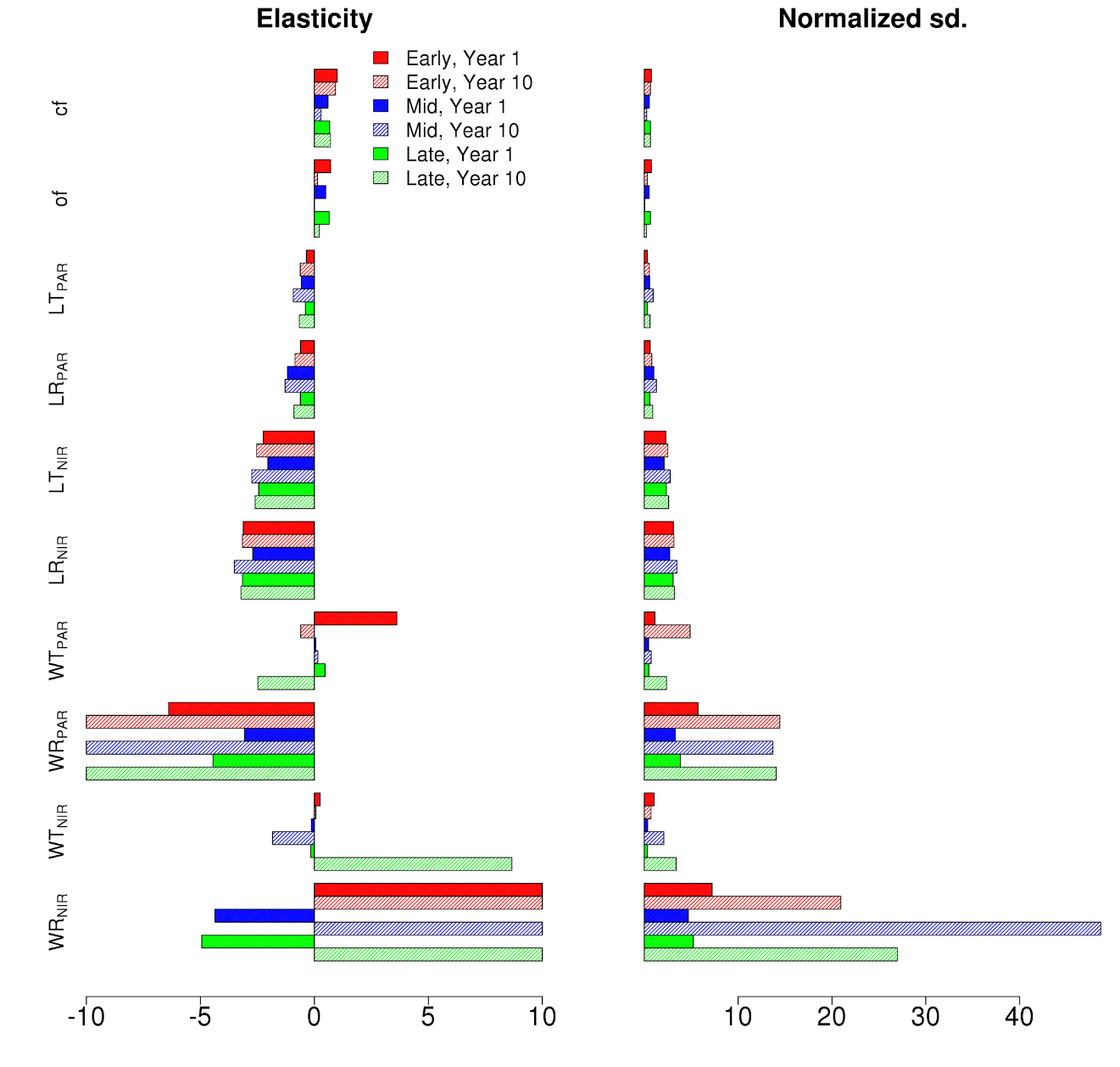


B)


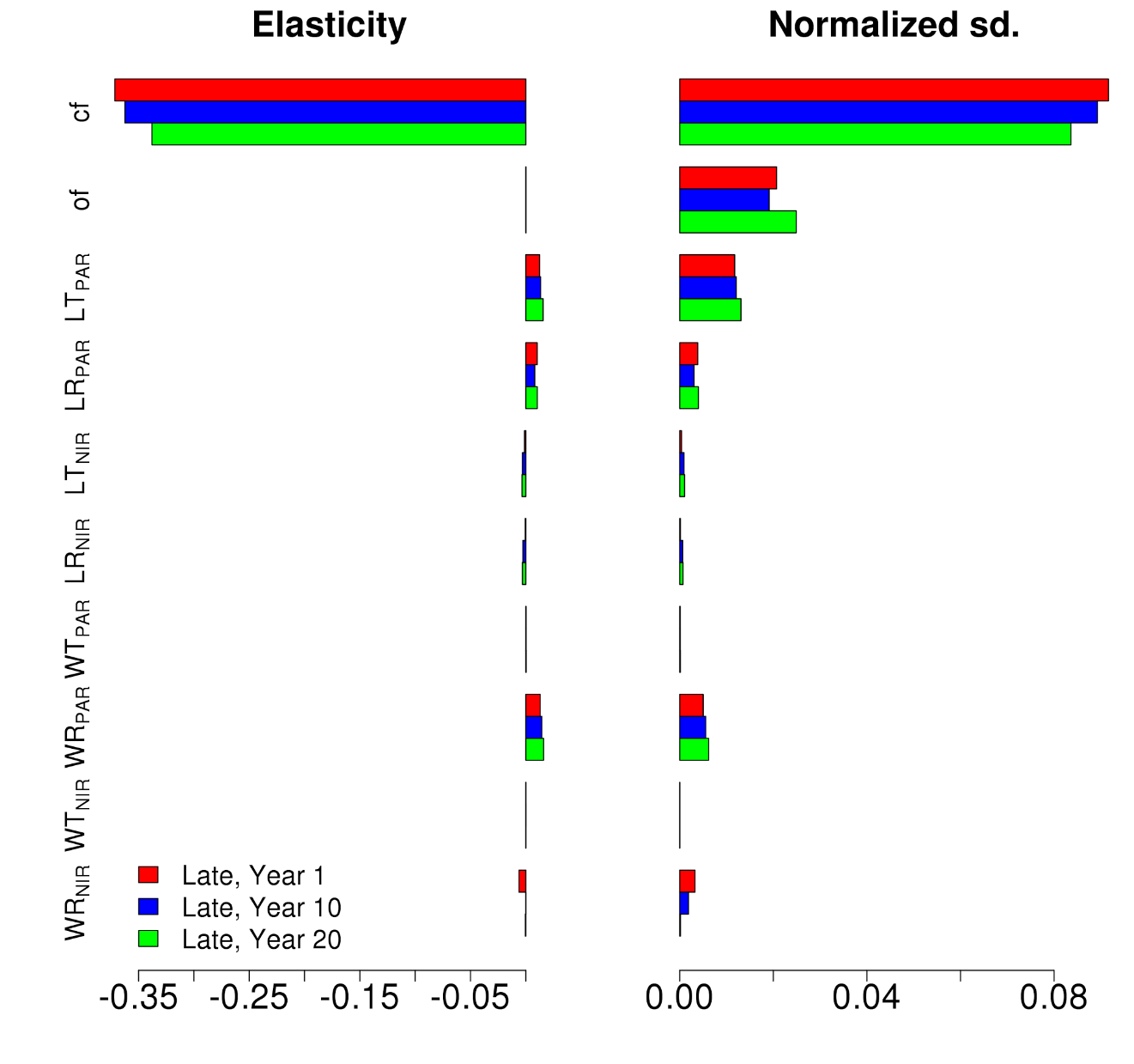


**C)**


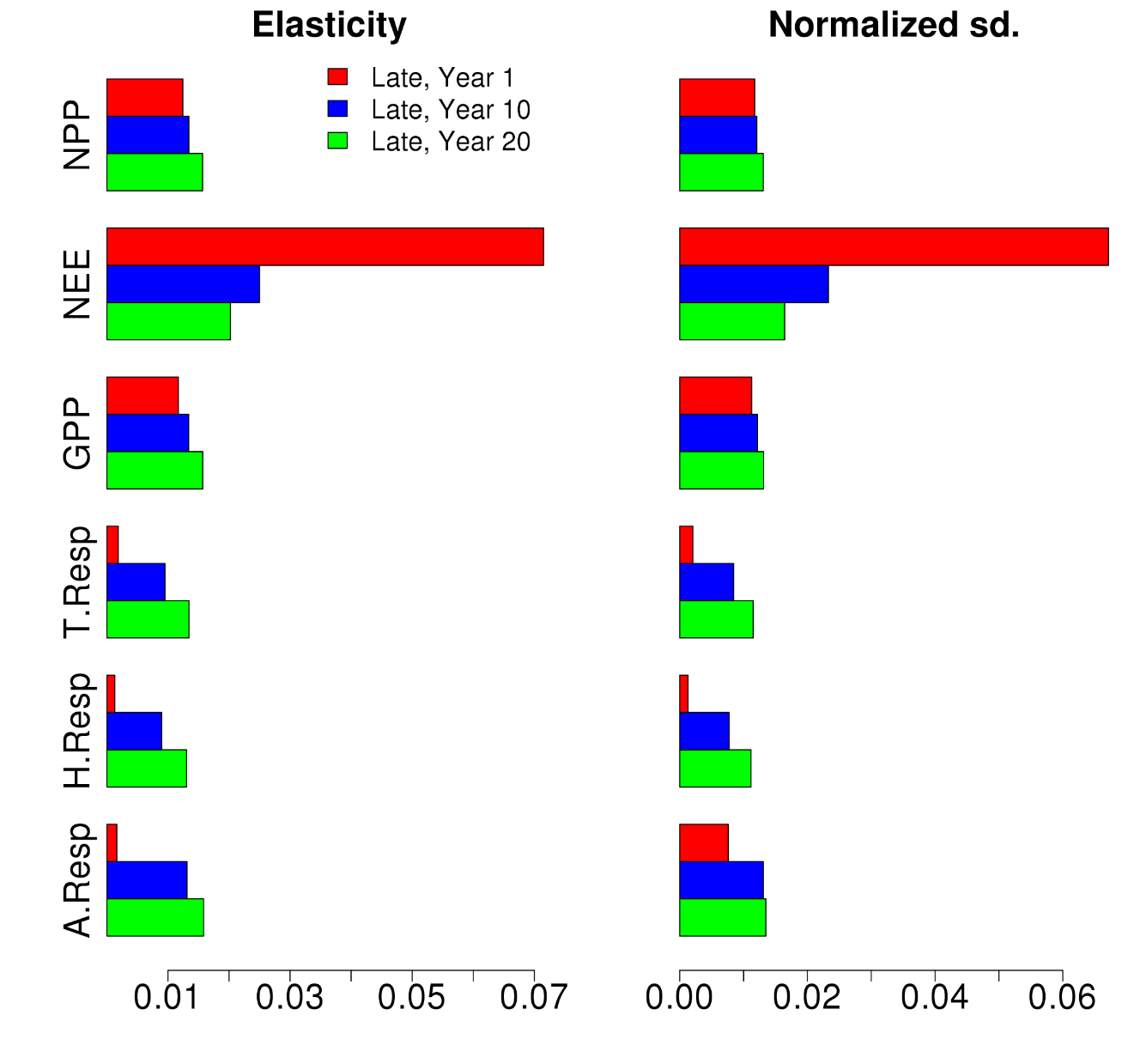

Supplement: S1 File — A) The Bowen Ratio elasticity and normalized output standard deviations of Early (Red), Mid (Blue) and Late (Green) Hardwood PFTs for the chosen radiative parameters. Results are shown for first (full) and tenth (shaded) year of the simulation. The elasticities were capped at 10 in order to show the variation across all parameters. B) The NPP elasticity and normalized output standard deviations for first (Red), tenth (Blue) and 20th (Green) years of simulation for Late Hardwood PFTs in the forest inventory canopy for the chosen radiative parameters. C) The variable elasticity and normalized output standard deviations for first (Red), tenth (Blue) and 20th (Green) years of simulation for Late Hardwood PFTs in the forest inventory canopy in response to changes in response to changes in Leaf PAR transmission. (DOCX) [file pone.0216512.s001.docx]
